# Association between sexual orientation acceptance and suicidal ideation, substance use, and internalised homophobia amongst the pink carpet Y cohort study of young gay, bisexual, and queer men in Singapore

**DOI:** 10.1186/s12889-021-10992-6

**Published:** 2021-05-22

**Authors:** Clarence Ong, Rayner Kay Jin Tan, Daniel Le, Avin Tan, Adrian Tyler, Calvin Tan, Chronos Kwok, Sumita Banerjee, Mee Lian Wong

**Affiliations:** 1 Saw Swee Hock School of Public Health, National University of Singapore, 12 Science Drive 2, MD1 Tahir Foundation Building #10-01, Singapore, 117549 Singapore; 2 Action for AIDS Singapore, 9 Kelantan Lane #03-01, Singapore, 208628 Singapore; 3 National University Hospital, National University Health System, Singapore, Singapore

**Keywords:** Sexual orientation, Homophobia, Internalized homophobia, Mental health, Men who have sex with men, Singapore, Coming out

## Abstract

**Background:**

Gay, bisexual and queer (GBQ) men are frequently subjected to minority stressors that have negative impacts on their health. Milestones that include the acceptance and disclosure of sexual identity amongst GBQ men are hence key instruments in understanding the prevalence of internalised homophobia and predicting health outcomes. As such, this work takes a novel approach to deduce the correlates of delayed acceptance of sexual orientation in young GBQ men as a measure of internalised homophobia through retrospective self-reporting and age-based analysis.

**Methods:**

Participants were recruited as part of a cohort study exploring the syndemic risks associated with HIV acquisition among young GBQ men in Singapore. We examined their levels of internalised, perceived, experienced homophobia, as well as their health behaviours and suicidal tendencies. Two separate variables were also self-reported by the participants – the age of questioning of sexual orientation and the age of acceptance of sexual orientation. We subsequently recoded a new variable, delayed acceptance of sexual orientation, by taking the difference between these two variables, regressing it as an independent and dependent variable to deduce its psychosocial correlates, as well as its association with other measured instruments of health.

**Results:**

As a dependent variable, delayed acceptance of sexual orientation is positively associated with an increase of age and internalised homophobia, while being negatively associated with reporting as being gay, compared to being bisexual or queer. As an independent variable, delayed acceptance of sexual orientation was associated with a delayed age of coming out to siblings and parents, suicide ideation, historical use of substances including smoking tobacco cigarettes and consuming marijuana, as well as reporting higher levels of experienced, internalised and perceived homophobia.

**Conclusion:**

Greater levels of early intervention and efforts are required to reduce the heightened experience of minority stress resulting from communal and institutional hostilities. Areas of improvement may include community-based counselling and psychological support for GBQ men, while not forsaking greater education of the social and healthcare sectors. Most importantly, disrupting the stigma narrative of a GBQ ‘lifestyle’ is paramount in establishing an accepting social environment that reduces the health disparity faced by GBQ men.

**Supplementary Information:**

The online version contains supplementary material available at 10.1186/s12889-021-10992-6.

## Background

Gay, bisexual and queer (GBQ) men constitute a key population that are disproportionately affected by sexual and mental health risks, with several studies having demonstrated significant health risk behaviours and depreciative health outcomes for GBQ men compared to their heterosexual peers [1–3]. This includes a greater incidence of mental health issues including suicidal tendencies [4–6] and non-suicidal self-injury [7, 8]. This may be due to their stigmatised identity within society, young GBQ men are prone to minority stress [9], which largely originate from negative experiences of discrimination, social rejection and abuse [4, 10]. As sexual minorities, young GBQ men are also more vulnerable to substance abuse [11] and prone to high-risk sexual behaviours leading to a higher incidence of sexually transmitted diseases [1, 12]. As such behaviours of self-harm may transcend into adulthood from adolescence [13], it is important to address these issues earlier in the life course of GBQ men.

Experienced, internalised and perceived homophobia are three main minority stressors that impact GBQ men. Internalised homophobia is the most proximal stressor to the individual, which involves the internalisation of heteronormative societal attitudes without direct experiences of rejection [14, 15]. Despite its intrapsychic nature, internalised homophobia is determined by the social climate [16, 17], and according to attachment theory, the quality of early experiences with attachment figures during their development [18, 19]. The society and security of attachments thus facilitate or hinder exploration and acceptance of one’s sexuality, thus affecting self-esteem [20] and support-seeking experiences [21]. Young GBQ men with high levels of internalised homophobia may therefore struggle with the management of negative emotions [22] and the management of stress and fear [23], which may lead to greater levels of anxiety and even suicide ideation [24, 25]. Moreover, a significant association is found between high levels of internalised homophobia with alcohol use disorder and substance abuse [26, 27], as well as small levels of sexual risk-taking behaviours [28].

Sexual identity development theory suggests that self-acceptance and coming out to others improves one’s positive sense of self and health [9, 29]. Some authors have also considered self-acceptance of sexual identity to be inversely related to internalised homophobia [30, 31]. Therefore, milestones of sexual identity and disclosure through self-report instruments may capture internalised homophobia, with a greater time between these milestones found to be positively associated with stress [32]. Fortunately, the time between these milestones is becoming shorter for younger cohorts [33], with the age of coming out decreasing by one year of age every two to five years [34]. In recent years, the median ages at which GBQ men start questioning their sexuality and the age they were sure are 12 and 15 respectively [35].

Most scholars have focused on the decreasing trends of these milestones across time [36, 37] or changes in sexual attraction amongst men [38, 39]. However, studies on changes in sexual identity have also mainly be limited to young women [40, 41]. Furthermore, there has been no available studies on the length of time between questioning and acceptance of one’s sexuality amongst GBQ men in highly stigmatised contexts. Prior studies have shown that individuals face considerable barriers to coming out in highly stigmatised context such as at work [42], or in the healthcare setting [43]. In countries such as China, 80% of GBQ men had not disclosed their sexual orientation to healthcare professionals [44]. Within the Singapore context that emphasises heteronormativity, clinics become costly spaces for disclosure of sexual orientation, leading to poorer testing behaviours and health outcomes [45]. Conversely, disclosure towards a non-LGBTQ family member is correlated with better testing behaviours and health outcomes [46].

Singapore society has largely held negative perceptions of, and attitudes towards lesbian, gay, bisexual, and transgender (LGBT) individuals [47]. Criminal legislation towards sexual minorities have corresponded, and arguably, contributed to these negative attitudes. Section 377A of the Singapore penal code criminalises consensual sexual behaviour between men, with penalties for imprisonment for a term that may extend up to no longer than two years; section 294 of the Singapore penal code has also criminalised sexual and non-sexual homosexual activities in public, which is regarded as an obscene act. Other forms of societal surveillance and discipline include the non-recognition of same-sex union, the regulation of the status of children in non-heteronormative couples, censorship of LGBTQ content in the media, and the lack of targeted laws specifically protecting LGBTQ Singaporeans against discrimination. According to Foucault [48], the social environment of such laws and policies have the impact of regulating the thoughts and behaviours of sexual minorities. With self-acceptance presupposing disclosure of sexual orientation, it may be inferred that the self-acceptance of GBQ men in Singapore is delayed, and thus inconsistent with studies within a Western context.

The impetus of this study is therefore two-fold. First, to investigate the correlates of delayed acceptance of sexual orientation, due to the lack of data on this in Singapore and its potential implications for health promotion interventions; and second, to explore the associations between delayed acceptance of sexual orientation of GBQ men with a variety of health outcomes. Specifically, this study hypothesises that internalised homophobia has a positive association with delayed acceptance of one’s sexual orientation, and tests this by exploring and adjusting for a variety of psychosocial correlates, including measures of homophobia, social capital, and outness, that may be associated with the acceptance of one’s sexual orientation. We then explore the delayed acceptance of one’s sexual orientation’s association with a variety of health outcomes relating to sexual and mental health.

## Methods

### Participants and recruitment

The Pink Carpet Y Cohort Study is a prospective cohort study exploring the syndemic risks associated with HIV and other sexually transmitted infections (STI) acquisition among YMSM in Singapore. This study was a partnership between Action for AIDS Singapore (AFA), one of Singapore’s longest-running community-based organizations serving the health of GBQ men, and the National University of Singapore (NUS). To be eligible for this cohort, participants had to be HIV-negative or unsure of their HIV status, between the ages of 18 to 25 years old, Singapore citizens or permanent residents, and identify as gay, bisexual, or queer men at the point of recruitment, which spanned across May to September 2019. Ethics approval was obtained from the institutional review board at the National University of Singapore (Reference Code S-19-007) prior to data collection.

Participants were invited to participate in this study through a network of community-based organizations in Singapore who are engaged in health advocacy-related activities for GBQ men. Participants who were interested in participating and were eligible for the study signed up through an enrolment link. An AFA staff member subsequently verified the eligibility of participants who had signed up prior to sending them a unique identifier, and a link to access the baseline survey.

To safeguard participants’ identities, the researchers ensured that no staff member from AFA or NUS had full access to either participants’ personal details held by AFA, and the baseline survey results held by NUS. Both sets of data were only linked by the unique identifier which participants entered at the beginning of the survey. Upon completion of the survey, a NUS staff member provided AFA with the unique identifiers who had completed the baseline survey, and an SGD20.00 (approximately USD15.00) cash reimbursement was given to the participant. Participants could also refer their friends to participate in the survey and be reimbursed SGD5.00 (approximately USD3.75) for each friend successfully referred and who had completed the baseline survey.

### Variable measures

A copy of the survey questionnaire developed for this study may be found in Supplementary File 1. The survey collected sociodemographic information from respondents, including age (in years), ethnicity (Chinese, Malay, Indian, Others), gender (cisgender, transgender, genderqueer, or others), sexual orientation (gay, bisexual, queer, others), HIV status (HIV-negative, unsure) and monthly household (in SGD dollars). Some collected sociodemographic information were subsequently recoded as a means of achieving sufficient subgroup sizes for the purposes of regression analyses for some variables. Ethnicity (Chinese or non-Chinese), gender (cisgender or non-cisgender), sexual orientation (gay or bisexual, queer and others), and monthly household income (Singapore Dollars [SGD] 5000 and above or below SGD5000; SGD5000 is approximately USD3668.94) were recoded as binary variables. The cut-off for household income was determined through the median gross personal income of Singapore residents [49]. As the GBQ men in our sample included respondents who were still in school, educational attainment and gross monthly personal income were omitted as variables, though they were collected in the baseline survey. Household income was chosen as a proxy variable for socioeconomic status among participants, given that most participants were not expected to report a personal monthly income that would reflect the socioeconomic strata in which they were embedded.

We collected several variables around an individual’s milestones and attitudes towards their personal sexual orientation, all of which were self-reported by participants. Firstly, the age of awareness of their same-sex attraction; secondly, the age of questioning of their sexual orientation; thirdly, the age of acceptance of sexual orientation. The outcome variable of interest, delayed acceptance of sexual orientation, was quantified by taking the difference between the age of questioning and age of acceptance of sexual identity. The age of awareness of same-sex attraction was not used due to too many missing values. For participants who inputted ‘Never’ or ‘NA’ for their value under the age of questioning variable, the difference was treated as zero; for participants who inputted a range of ages for their value under the age of questioning variable, the lower value was used. The current age of the participant was used for one participant that had inputted ‘NIL’ as a value under the age of acceptance, as it was assumed that they have yet to accept their sexual identity. Responses where the age of questioning was reported to be after the age of acceptance (*n* = 6) were removed on the basis that participants had not interpreted the question correctly.

We collected a range of exposure variables that included a range of variables that we thought were epidemiologically relevant to the outcome variable based on our literature review. Depression severity was measured through the well-established, nine-item patient health questionnaire-9 (PHQ-9) validated by Kroenke and colleagues [50, 51]. Depression severity was measured as an index that was the sum score of all nine items, with a minimum score of 0 and a maximum score of 27. Cronbach’s alpha of the scale was reported as 0.92. Connectedness to the LGBT community was an eight-item scale adapted from Frost and Meyer [52]. Community connectedness was measured as an index that was the sum score of all eight items, with a minimum score of 8 and a maximum score of 32. Cronbach’s alpha of the scale was reported as 0.86. Outness was measured through the outness inventory, a 10-item scale developed by Mohr and Fassinger [53]. The outness inventory assesses the degree or magnitude to which lesbian, gay, and bisexual (LGB) individuals are open or ‘out’ about their sexual orientation to other individuals. The overall outness score was calculated as an average of three subscales, including *outness to family*, *outness to religion*, and *outness to the world*. Cronbach’s alpha of the scale was reported as 0.82. Bonding social capital was measured through the brief 16-item personal social capital scale 16 (PSCS-16) validated by Wang and colleagues [54, 55]. The first eight items on the PSCS-16 were designed to measure bonding social capital, while the last eight items measured bridging social capital. Cronbach’s alpha of 0.77 was reported for the bonding social capital subscales.

Experienced homophobia was a 14-item scale developed by Ramirez-Valles and colleagues [56]. It assesses the degree or magnitude to which GBQ men experienced stigma and discrimination for their sexual identity growing up and in adulthood. Internalised homophobia was a five-item scale developed by Amola and Grimmett [57]. It assesses the degree or magnitude to which GBQ men have negative perceptions of their own sexual identity. Perceived homophobia was a six-item scale developed by Smolenski, Ross, Risser and Rosser [58]. It assesses the general perception to which GBQ men feel towards the GBQ community at large. Cronbach’s alpha for these scales were reported as 0.90, 0.84 and 0.82, respectively.

We also collected a range of social factors and health outcomes that we wanted to explore associations with the outcome variable. These included HIV prevention variables such as testing and knowledge of HIV post-exposure prophylaxis and pre-exposure prophylaxis, age-related factors such as age of sexual debut and age of coming out, suicide indicators such as ever contemplating suicide (suicide ideation) or attempting suicide (suicide attempts), and substance use variables, which included a series of identical questions that solicited a yes vs no response from participants for a series of different recreational or illicit substances in the context of Singapore, including smoking of tobacco cigarettes, alcohol, marijuana, amyl nitrites (or poppers), methamphetamine and Gamma Hydroxybutyrate and Gamma-Butyrolactone (GHB/GBL).

### Data analysis

Statistical analysis was carried out using the statistical software STATA version 16 (Stata Corp, College Station, TX, USA). We employed descriptive statistics to identify trends in sample characteristic; reporting median and interquartile ranges as well as means and standard deviations for non-normally and normally-distributed data, respectively. We conducted two sets of analyses; first, to assess the demographic and psychosocial correlates of delayed acceptance; and second, to assess how delayed acceptance is a factor to a range of health-related variables. This was done because of the policy-relevance of understanding who among YMSM might be susceptible to delayed acceptance, and at the same time investigate the health outcomes that are associated with delayed acceptance. While bivariable and multivariable linear and Poisson regression models with robust sandwich variances were used to compute the crude coefficient (C), prevalence ratio (PR), adjusted coefficient (aC) and adjusted prevalence ratio (aPR) with delayed acceptance of sexual orientation as both a dependent and independent variable. Statistical significance was set at *p* < 0.05.

## Results

### Sociodemographic attributes and description of the analytic sample

A total of 570 participants were recruited in this study, and 564 remained in the analytic sample after removing six responses based on their lack of understanding of the questions pertaining to the outcome variable of interest. In terms of their sociodemographic attributes, the mean age of the sample was 21.9 years (SD = 2.17). 83.7% of the participants identified as Chinese (*n* = 472), 92% identified as cisgender male (*n* = 519), 71.6% identified as gay (*n* = 404), and 35.8% reported a monthly household income of SGD5000 and above (*n* = 202). The mean age of the participants’ internal awareness of same-sex attraction was 13.3 (SD = 3.2), the mean age of questioning of sexual orientation was 13.7 (SD = 3.08), and the mean age of acceptance towards their sexual orientation was 16.6 (SD = 3.03). Taking the difference between the participants’ age of questioning and age of acceptance of sexual orientation, the median year gap was 2 (IQR = 3).

Participants also scored a median of 6 (IQR = 10) for depression severity based on the Patient-Health Questionnaire-9, and had an outness inventory of 2.25 (IQR = 2) for the outness scale. An average score of 22.4 (SD = 4.33) was reported for the connectedness to the LGBT community scale and an average score of 21.7 (SD = 4.91) was reported for the bonding social capital subscale. For the indicators of homophobia, the median score of experienced homophobia was 25 (IQR = 12), the mean score for internalised homophobia was 10.4 (SD = 3.76), and the mean score for perceived homophobia was 17.7 (SD = 3.41). Table 1 summarises the sociodemographic attributes and overall description of the analytic sample.

**Table 1:** Sociodemographic attributes and description of analytic sample (*n* = 564)

Demographic Variables	n	%	Mean	SD	Median	IQR
**Age**			21.9	2.17		
**Ethnicity**						
Chinese	472	83.7%				
Non-Chinese	92	16.3%				
**Gender**						
Cisgender male	519	92.0%				
Transgender, genderqueer, or others	45	8.0%				
**Sexual orientation**						
Gay	404	71.6%				
Bisexual, queer, or others	160	28.4%				
**HIV status**						
HIV-negative	475	84.2%				
Unsure	89	15.8%				
**Monthly household income**						
SGD 5000 and above	202	35.8%				
Below SGD 5000	362	64.2%				
**Attitudes towards sexual orientation**						
Age of awareness of same-sex attraction			13.3	3.2		
Age of qeuestioning sexual orientation			13.7	3.08		
Age of acceptance towards sexual orientation			16.6	3.03		
Difference in age of questioning and age of acceptance					2	3
**Depression severity (PHQ-9)**					6	10
**Outness inventory**					2.25	2
**Connectedness to LGBT community**			22.4	4.33		
**Bonding social capital**			21.7	4.91		
**Experienced homophobia**					25	12
**Internalised homophobia**			10.4	3.76		
**Perceived homophobia**			17.7	3.41		

*Abbreviation*: *SD* Standard Deviation, *IQR* Interquartile Range, *PHQ-9* Patient Health Questionnaire 9

### Factors associated with delayed acceptance of sexual orientation

A summary of factors associated with delayed acceptance of sexual orientation may be found in Table 2. At the bivariable level, age (C = 0.15, 95%CI [0.05–0.25]) was positively associated with delayed acceptance of sexual orientation amongst the participants; in other words. Furthermore, experienced homophobia (C = 0.032, 95%CI [0.006–0.058]), internalised homophobia (C = 0.095, 95%CI [0.038–0.153]) and perceived homophobia (C = 0.075, 95%CI [0.011–0.138]) were also positively associated with delayed acceptance of sexual orientation amongst the participants.

**Table 2:** Multivariable linear regression for difference between age of acceptance and questioning

	Difference between age of acceptance and questioning (n = 564)							
	C	95% CI		***p***-value	aC	95% CI		p-value
Age	0.15	0.05	0.25	**0.003**	**0.15**	**0.05**	**0.25**	**0.004**
Non-Chinese (ref = Chinese)	−0.08	− 0.67	0.51	0.786	−0.07	− 0.68	0.54	0.821
Cisgender male (ref = Transgender, genderqueer, or others)	−0.64	−1.44	0.17	0.120	**−0.85**	**−1.68**	**− 0.02**	**0.046**
Gay (ref = Bisexual, queer, or others)	0.13	−0.35	0.61	0.600	0.21	−0.30	0.72	0.417
> SGD5000 household income (ref = SGD5000 or less)	0.23	−0.23	0.68	0.327	0.19	−0.28	0.66	0.423
Depression severity (PHQ-9)	0.01	−0.03	0.04	0.693	−0.01	−0.05	0.02	0.505
Outness inventory	0.03	−0.13	0.18	0.742	0.05	−0.14	0.23	0.613
Connectedness to LGBT community	0.00	−0.05	0.05	0.904	0.00	−0.05	0.05	0.971
Bonding social capital	−0.01	−0.05	0.04	0.741	−0.01	−0.06	0.04	0.639
Experienced homophobia	**0.03**	**0.01**	**0.06**	**0.013**	0.02	0.00	0.05	0.087
Internalised homophobia	**0.10**	**0.04**	**0.15**	**0.001**	**0.10**	**0.03**	**0.16**	**0.003**
Perceived homophobia	**0.07**	**0.01**	**0.14**	**0.021**	0.04	−0.03	0.11	0.254

Notes

*Abbreviation*: *CI* Confidence Interval, *C* Coefficient, *aC* Adjusted Coefficient, *SGD* Singapore Dollars, *PHQ-9* Patient Health Questionnaire 9

At the multivariable level, analyses revealed that after controlling for all covariates in the model, age (aC = 0.15, 95%CI [0.05–0.25]) remained positively associated with delayed acceptance of sexual orientation amongst the participants. On the other hand, cisgender males (aC = − 0.85, 95%CI [− 1.68--0.02]), as compared to transgender and genderqueer, are negatively associated with delayed acceptance of sexual orientation amongst the participants. While experienced homophobia and perceived homophobia were no longer significant, internalised homophobia (aC = 0.097, 95%CI [0.033–0.160]) remained positively associated with delayed acceptance of sexual orientation amongst the participants.

### Social outcomes and health behaviours associated with delayed acceptance of sexual orientation

A summary of social outcomes and health behaviours associated with delayed acceptance of sexual orientation may be found in Table 3. At the bivariable level, delayed acceptance of sexual orientation as an independent variable is positively associated with delayed coming out to siblings (C = 0.122, 95%CI [0.031–0.214]) and coming out to parents (C = 0.155, 95%CI [0.069–0.242]) amongst the participants. Moreover, delayed acceptance of sexual orientation is also positively associated with suicide ideation (PR = 1.036, 95%CI [1.011–1.060]). In terms of substance consumption, delayed acceptance of sexual orientation is positively associated with smoking (PR = 1.049, 95%CI [1.012–1.088]) and marijuana usage (PR = 1.109, 95%CI [1.026–1.197]). For the indicators of homophobia, delayed age of acceptance is positively associated with experienced homophobia (C = 0.340, 95%CI [0.077–0.312], internalised homophobia (C = 0.195, 95%CI [0.077–0.312]) and perceived homophobia (C = 0.126, 95%CI [0.019–0.233]).

**Table 3:** Multivariable linear & poisson regression with difference between age of acceptance and questioning as independent variable

	Difference between age of acceptance and questioning as independent variable (n = 564)							
	C/PR	95% CI		p-value	aC/aPR^a^	95% CI		p-value
**HIV Prevention**								
HIV Status Unknown (ref = Status Known)	0.98	0.91	1.06	0.647	1.00	0.93	1.08	0.959
HIV Never Tested (ref = Ever Tested)	1.01	0.96	1.05	0.759	1.02	0.98	1.06	0.395
HIV Test Irregularity (ref = Regular)	0.97	0.91	1.03	0.331	0.96	0.90	1.03	0.256
STI Test Irregularity (ref = Regular)	0.99	0.95	1.02	0.458	1.00	0.96	1.03	0.792
Knowledge of PrEP (ref = No Knowledge)	1.00	0.99	1.02	0.794	1.00	0.98	1.01	0.802
Knowledge of PEP (ref = No Knowledge)	1.00	0.97	1.02	0.778	0.99	0.97	1.01	0.362
**Aged Related Behaviour**								
Age of Sexual Debut	0.07	−0.03	0.16	0.168	−0.21	−0.91	0.49	0.562
Coming out to Sibling(s)^b^	**0.12**	**0.03**	**0.21**	**0.008**	**0.13**	**0.04**	**0.23**	**0.004**
Coming out to Parent(s)^b^	**0.16**	**0.07**	**0.24**	**0.000**	**0.17**	**0.08**	**0.25**	**0.000**
Coming out to Peer(s)^b^	0.06	−0.04	0.16	0.232	0.08	−0.02	0.18	0.130
**Suicide Indicator**								
Suicide Ideation (ref = No Suicide Ideation)	**1.04**	**1.01**	**1.06**	**0.004**	**1.04**	**1.02**	**1.07**	**0.001**
Suicide Attempt (ref = No Suicide Attempt)	0.96	0.88	1.04	0.297	0.96	0.88	1.04	0.325
**Substance Consumption Behaviour**								
Smoking (ref = Never Smoked)	**1.05**	**1.01**	**1.09**	**0.008**	**1.05**	**1.01**	**1.09**	**0.011**
Alcohol (ref = Never had Alcohol)	1.00	0.99	1.02	0.503	1.00	0.99	1.02	0.588
Marijuana (ref = Never had Marijuana)	**1.11**	**1.03**	**1.20**	**0.009**	**1.06**	**1.00**	**1.12**	**0.043**
Poppers (ref = Never had Poppers)	1.00	0.96	1.06	0.854	0.99	0.95	1.04	0.744
Methamphetamine (ref = Never had methamphetamine)	1.00	0.86	1.17	0.979	0.99	0.85	1.15	0.897
GHB/GBL (ref = Never had GHB/GBL)	0.94	0.83	1.08	0.377	0.93	0.82	1.07	0.304
**Social Indicator**								
Depression severity (PHQ-9)	0.04	−0.17	0.25	0.693	0.06	−0.15	0.27	0.581
Outness inventory	0.01	−0.04	0.05	0.742	0.00	−0.05	0.04	0.915
Connectedness to LGBT community	−0.01	−0.14	0.13	0.904	−0.01	−0.15	0.13	0.898
Bonding social capital	−0.03	−0.18	0.13	0.741	−0.04	−0.19	0.11	0.608
Experienced homophobia	**0.34**	**0.07**	**0.61**	**0.013**	**0.33**	**0.07**	**0.60**	**0.014**
Internalised homophobia	**0.19**	**0.08**	**0.31**	**0.001**	**0.21**	**0.09**	**0.32**	**0.000**
Perceived homophobia	**0.13**	**0.02**	**0.23**	**0.021**	**0.13**	**0.03**	**0.24**	**0.016**

Notes

*Abbreviation*: *CI* Confidence Interval, *C* Coefficient, *aC* Adjusted Coefficient, *PR* Prevalence Ratio, *aPR* Adjusted Prevalence Ratio, *GHB/GBL* Gamma hydroxybutyrate/Gamma butyrolactone, *PHQ-9* Patient Health Questionnaire 9

Prevalence ratio is presented for dependent variables with references while the coefficient is presented for depedent variables without

^a^Adjusted for age, ethnicity, gender, sexuality and income

^b^Adjusted values excludes age as a variable

At the multivariable level, outcomes were controlled with the sociodemographic attributes of the participants, although the age variable was excluded for age-related behaviour to avoid multicollinearity. Delayed acceptance of sexual orientation remained positively associated with delayed coming out to siblings (aC = 0.135, 95%CI [0.044–0.225]) and coming out to parents (aC = 0.166, 95%CI [0.080–0.253]) amongst the participants. Moreover, delayed age of acceptance also remained positively associated with suicide ideation (aPR = 1.040, 95%CI [1.015–1.065]). In terms of substance consumption, delayed acceptance of sexual orientation remained positively associated with smoking (aPR = 1.050, 95%CI [1.011–1.090]) and marijuana usage (aPR = 1.061, 95%CI [1.002–1.124]). Lastly, for the indicators of homophobia, delayed acceptance of sexual orientation also remained positively associated with experienced homophobia (aC = 0.332, 95%CI [0.068–0.596], internalised homophobia (aC = 0.208, 95%CI [0.092–0.324]) and perceived homophobia (aC = 0.134, 95%CI [0.025–0.242]).

## Discussion

This study sought to identify the correlates of delayed acceptance of sexual orientation amongst young GBQ men, as well as its association with social outcomes and health behaviours. These findings provide a means of discussing and hypothesising propensities of cognitive dissonance amongst young GBQ men resulting from incongruent inward prejudice against their sexual orientation and their sexual attraction. It also provides data that allows for an assessment of areas of intervention to improve social and health outcomes.

We found that an increase in age is associated with an increased difference between the age of questioning and age of acceptance of sexual orientation. We hypothesise that this may be attributable to a time gap associated with improved societal attitudes towards homosexuality [59–61] and greater representation of GBQ men in media and healthcare in recent years [62, 63]. This results in the reduced risk of coming out, therefore resolving the internal conundrums of sexual orientation amongst GBQ men. Younger GBQ men, who are more likely to grow up in a GBQ friendly cultural context, may thus report a reduced gap in the age of questioning and acceptance of their sexual orientation. Not surprisingly, cisgender GBQ men are less likely to exhibit greater delayed acceptance of their sexual orientation. Potential changes in sexual orientation to reflect the emerging identity of transgender individuals after transitioning has been reported [64] and is in fact quite common between both transgender men and women [65]. These realities, usually predisposed and accompanied by the lack of structural support for transgender and genderqueer individuals, may exacerbate internal conflicts of sexual identity, resulting in greater turbulent years of questioning of sexual orientation.

Internalised homophobia naturally aggravates the struggle of acceptance of sexual orientation amongst young GBQ men. The cognitive dissonance fuelled by heteronormative structures and environments may lead to issues of self-hatred, shame and anxiety among young GBQ men who report higher levels of internalised homophobia [24, 25], which delays self-acceptance as they stumble to overcome the misdirected anger towards themselves. Although experienced and perceived homophobia were only statistically significant at a bivariable level, their impact on the self-acceptance of sexual orientation amongst GBQ men should be not ignored. A Pearson correlation test revealed a modest association between experienced and perceived homophobia (r = 0.2418, *p* = 0), and internalised and perceived homophobia (r = 0.2157, p = 0).

Another key finding of this study is that delayed acceptance of sexual orientation is positively associated with suicide ideation. This finding corroborates the minority stress theory [9, 14] which posits that sexual minorities are more prone to chronic stressors related to their sexual identity; in other words, the internalisation of negative social attitudes compromises the mental health and well-being of GBQ men, which propels the ideation of self-harm and suicide. While a history of suicide attempt is not statistically significant in this study, there is a moderate association between suicide ideation and suicide attempt (r = 0.3594, *p* = 0.000). This suggests suicide ideation is a strong condition of suicide attempt, and further study is necessary to explore the intersection between these two factors. Delayed acceptance of sexual orientation was also found to be associated with a delay in the age of coming out to parents and siblings. This is unsurprising given that self-acceptance is likely to precede coming out and sharing of one’s formed identity with others.

While exploring behaviours of substance usage, delayed acceptance of sexual orientation was found to be positively associated with smoking and usage of marijuana. These findings do not necessarily indicate substance dependence, but supports a wider literature that puts GBQ men with negative beliefs and values of their sexual identity as having a higher risk of substance use [26, 27]. Using our age-based approach towards internalised homophobia, our findings validate Kus’ theory [66] of how substance is used to cope to stress between stages of identification and cognitive change. It also supports Lu and Shaham’s study [67] on the association of self-administration of substances with various types of stressors. While a vast literature demonstrates a higher prevalence of smoking amongst sexual minorities [68–70], social influence from parents and peers [71] and its coincidence with the development period in the midst of emerging adulthood should also be taken into account [72]. Furthermore, further analysis shows a weak association (r = 0.0530, *p* = 0.209) between smoking and delayed acceptance.

The consistent statistical significance of indicators of homophobia associated with delayed acceptance of sexual orientation highlights their mutually reinforcing capacities. This vicious cycle reveals a negative compounding effect of homophobia on self-acceptance, underpinning the key role homophobia plays in health outcomes. We hypothesise that sensitivities to homophobia are heightened amongst GBQ men experiencing turbulent years of acceptance, thus suggesting the importance of social networks and cultural acceptance in the formulation of their sexual identity. This therefore stresses the need for early intervention on an institutional and communal level to ensure greater health outcomes for such sexual minorities.

These findings on the association between sexual orientation acceptance and suicidal ideation, substance use, and internalised homophobia reflect the tragedies that are associated with invoking the myth of “Asian sexuality” in Singapore [73]. In constructing a rhetoric where Singaporeans are opposed to homosexuality, GBQ men are seen as the deviant ‘other’, accentuating their minority consciousness. This phenomenon is reflective of Foucault’s concept of biopower, where the state subjugates and controls the bodies of the population and hence public health [74]. Sexual orientation and behaviours thus become objects of societal moral concern, justifying the government’s repressive sexual policies against GBQ men. In this sense, our findings are inconsistent with the minority resilience hypothesis that acknowledges “the power minority groups have with respect to prejudice” [75]. Such a form of viewing GBQ men is largely meant to preserve a Western view that “emphasises control, freedom, and individualised determination” [76] and fails to reflect Singapore as a society. Our research hence supports an objective view of stress (i.e. prejudice event) that is defined by real and observable phenomena that are experienced. This allows us to reduce the risk of viewing the failure of GBQ men to cope with stress as a personal and subjective issue, but rather one that is attributed to societal failings and stressors that require intervention and abolishment respectively.

While this study employs the difference between the age of questioning and acceptance as an alternative continuous indicator for internalised homophobia, we remain mindful of certain limitations within this study. The age of questioning and acceptance is self-reported by participants, with the retrospective cross-sectional approach prone to memory bias which impairs participants’ ability to recall with accuracy. This may hence result in a smaller than expected difference calculated, as participants input the same age for both fields for convenience sake. Furthermore, as drug use carries severe penalties in Singapore, participants may not be entirely honest with their answers around drug use, which may have led to an underreporting of methamphetamine use in the present sample. This form of non-differential misclassification would have biased our results towards the null.

## Conclusions

Our study investigated how a delay in acceptance of one’s sexual identity is indicative of internalised homophobia, while also investigating its possible origins and impacts on social and health outcomes. It uncovered the negative reinforcing capacity of homophobia on delayed acceptance of sexual orientation and its psychosociological effects that result in substance usage and suicide ideation amongst GBQ men. Nonetheless, the study has made clear the positive influence early intervention may provide for young GBQ men struggling with their sexual identity. Moving forward, we must explore solutions that target not just individuals, but society as a whole - to leverage on the interplay between these two dynamics.

Hostilities towards the GBQ community still exist on an institutional and communal level in Singapore. From the criminalisation of sexual relations between men to the misunderstandings towards the community, many GBQ men are disproportionately represented and suffer from health disparities. Efforts should hence be taken to reduce the heightened experiences of minority stress and barriers towards accessing social support. This includes, but are not limited to, intervention that focuses on suicide awareness and integrated services that provide community-based counselling and psychological support to GBQ struggling with their sexual identity. Through such means, maladaptive coping behaviours such as substance use may be detected early, with adequate resources dedicated to helping GBQ men through such turbulent times. Of course, this has to be promoted in tandem with a more informed social and healthcare sector to provide much-needed services to GBQ men without stigma or prejudice.

The stigma narratives by the populace towards the GBQ community also has to be disrupted. The lack of understanding by Singaporeans at large contribute to minority stress amongst GBQ men, inhibiting individuals from self-acceptance and coming out free from communal backlash. Abiding by the ethos of the family as a basic unit of society, Singapore must no longer see the ‘lifestyles’ of sexual minorities as incongruent to family values, while disregarding the narrative’s impact on the souring of family relations. Therefore, efforts should be made to provide parents and families with support on how to healthily negotiate coming out experiences amongst their children. A more informed society through better representation of GBQ men in the media and a non-conservative sexual education should be provided so internalised, experienced and perceived homophobia may be reduced amongst young GBQ men and Singaporeans. With a more accepting social environment in Singapore, we may start to bridge the inequalities of health outcomes and facilitate an inclusive society.

## Supplementary Information

**Additional file 1: Supplementary file 1**. Survey questionnaire, Copy of survey questionnaire developed for data collection in the study.

## Data Availability

The datasets used and/or analysed during the current study are available from the corresponding author on reasonable request.

## Abbreviations

GBQ: Gay, Bisexual and Queer
STI: Sexually Transmitted Infections
AFA: Action for AIDS Singapore
NUS: National University of Singapore
SGD: Singapore Dollars
GHB/GBL: Gamma Hydroxybutyrate and Gamma-Butyrolactone
C: Coefficient
PR: Prevalence Ratio
