# Supplementary material for: Association between sexual orientation acceptance and suicidal ideation, substance use, and internalised homophobia amongst the pink carpet Y cohort study of young gay, bisexual, and queer men in Singapore
Source: BMC Public Health. 2021 May 22;21:971. doi: 10.1186/s12889-021-10992-6 (PMC8141135; doi:10.1186/s12889-021-10992-6)
Supplement: Supplementary file 1 — Additional file 1: Supplementary file 1. Survey questionnaire, Copy of survey questionnaire developed for data collection in the study. [file 12889_2021_10992_MOESM1_ESM.docx]

**Supplementary File 1: Survey Questionnaire**

| **#** | **Question** | **Response categories** | | | | | | | | | | | | | | | | | |
| --- | --- | --- | --- | --- | --- | --- | --- | --- | --- | --- | --- | --- | --- | --- | --- | --- | --- | --- | --- |
|  | How old are you? | [Open-ended response] | | | | | | | | | | | | | | | | | |
|  | What is your gender? | Female  Male  Others: ___________________ | | | | | | | | | | | | | | | | | |
|  | What sexual orientation do you identify yourself with the most? | Straight / Heterosexual  Gay / Homosexual  Bisexual  Others: ___________________ | | | | | | | | | | | | | | | | | |
|  | What is your HIV status? | I am HIV-positive  I am HIV-negative  I am unsure of my HIV status | | | | | | | | | | | | | | | | | |
|  | What is your residence status? | **Please select all that apply:**  Singaporean citizen  Singapore permanent resident  Others (please specify): ________________________ | | | | | | | | | | | | | | | | | |
|  | How do you identify yourself ethnically? | Chinese  Malay  Indian  Others (please specify): *________________________* | | | | | | | | | | | | | | | | | |
|  | What is your religion? | Buddhism  Islam  Hinduism  Christianity  Taoism | | | | | | | | Sikhism  Agnostic  Atheist  Others (Please specify):  _____________________ | | | | | | | | | |
|  | What is the highest level of education you have completed? | Primary Education  Secondary Education  GCE ‘N’ Levels or equivalent  GCE ‘O’ Levels or equivalent  GCE ‘A’ Levels or equivalent  Professional Certificate  Diploma  Bachelor’s Degree  Postgraduate Degree  Others _(10)_ (please specify): *________________________* | | | | | | | | | | | | | | | | | |
|  | Are you currently working? | Employed, please specify job:  Unemployed  Retired  Student  Other, please specify: | | | | | | | | | | | | | | | | | |
|  | What type of housing are you staying in? | 1 Room HDB  2 Room HDB  3 Room HDB  4 Room HDB  5 Room HDB  Maisonette or other executive public housing  Condominium  Terrace, Bungalow, and other Private Landed Property  Others: ___________________ | | | | | | | | | | | | | | | | | |
|  | On average, what is your gross personal monthly income (before CPF and tax deductions, if any)? | Not earning an income  < SGD1000  SGD1000 – SGD1999  SGD2000 – SGD2999  SGD3000 – SGD3999  SGD4000 – SGD4999  SGD5000 and above | | | | | | | | | | | | | | | | | |
|  | At what age did you become aware that you were sexually attracted to individuals of the same sex? | [Open-ended response] | | | | | | | | | | | | | | | | | |
|  | At what age did you begin to question your sexual orientation? | [Open-ended response] | | | | | | | | | | | | | | | | | |
|  | At what age did you accept that you were gay, bisexual, or queer? | [Open-ended response] | | | | | | | | | | | | | | | | | |
| **Connectedness to LGBT Community Scale (Adapted from Frost & Meyer, 2012)** | | | | | | | | | | | | | | | | | | | |
|  | **These are questions about the LGBT community in Singapore. By LGBT community, we don't mean any particular group, but in general the wider community of LGBTQ people.** | **Strongly Disagree** | | | | **Disagree** | | | | | **Agree** | | | | **Strongly Agree** | | | | |
|  | You feel you are a part of Singapore’s LGBT community |  | | | |  | | | | |  | | | |  | | | | |
|  | Participating in Singapore’s LGBT community is a positive thing for you |  | | | |  | | | | |  | | | |  | | | | |
|  | You feel a bond with the LGBT community |  | | | |  | | | | |  | | | |  | | | | |
|  | You are proud of Singapore’s LGBT community |  | | | |  | | | | |  | | | |  | | | | |
|  | It is important for you to be politically active in Singapore’s LGBT community |  | | | |  | | | | |  | | | |  | | | | |
|  | If we work together, LGBT people can solve problems in Singapore’s LGBT community |  | | | |  | | | | |  | | | |  | | | | |
|  | You really feel that any problems faced by Singapore’s LGBT community are also your own problems |  | | | |  | | | | |  | | | |  | | | | |
|  | You feel a bond with other gay, bisexual, or queer men |  | | | |  | | | | |  | | | |  | | | | |
| **Personal Social Capital Scale (Adapted from Chen et al. 2012)** | | | | | | | | | | | | | | | | | | | |
|  | **How would you describe the number of people that you have in each of the following six categories?** | **A lot** | | | **More than average** | | | | **Average** | | | | **Less than average** | | | | **A few** | | |
|  | Your family members |  | | |  | | | |  | | | |  | | | |  | | |
|  | Your relatives |  | | |  | | | |  | | | |  | | | |  | | |
|  | Your neighbours |  | | |  | | | |  | | | |  | | | |  | | |
|  | Your friends |  | | |  | | | |  | | | |  | | | |  | | |
|  | Your coworkers/colleagues |  | | |  | | | |  | | | |  | | | |  | | |
|  | Your friends in the LGBT community |  | | |  | | | |  | | | |  | | | |  | | |
|  | **With how many of people in each of the following categories do you keep a routine contact?** | **All** | | | **Most** | | | | **Some** | | | | **Few** | | | | **None** | | |
|  | Your family members |  | | |  | | | |  | | | |  | | | |  | | |
|  | Your relatives |  | | |  | | | |  | | | |  | | | |  | | |
|  | Your neighbours |  | | |  | | | |  | | | |  | | | |  | | |
|  | Your friends |  | | |  | | | |  | | | |  | | | |  | | |
|  | Your coworkers/colleagues |  | | |  | | | |  | | | |  | | | |  | | |
|  | Your friends in the LGBT community |  | | |  | | | |  | | | |  | | | |  | | |
|  | **Among the people in each of the following six categories, how many can you trust?** | **All** | | | **Most** | | | | **Some** | | | | **Few** | | | | **None** | | |
|  | Your family members |  | | |  | | | |  | | | |  | | | |  | | |
|  | Your relatives |  | | |  | | | |  | | | |  | | | |  | | |
|  | Your neighbours |  | | |  | | | |  | | | |  | | | |  | | |
|  | Your friends |  | | |  | | | |  | | | |  | | | |  | | |
|  | Your coworkers/colleagues |  | | |  | | | |  | | | |  | | | |  | | |
|  | Your friends in the LGBT community |  | | |  | | | |  | | | |  | | | |  | | |
|  | **Among people in each of the following six categories, how many will definitely help you upon your request?** | **All** | | | **Most** | | | | **Some** | | | | **Few** | | | | **None** | | |
|  | Your family members |  | | |  | | | |  | | | |  | | | |  | | |
|  | Your relatives |  | | |  | | | |  | | | |  | | | |  | | |
|  | Your neighbours |  | | |  | | | |  | | | |  | | | |  | | |
|  | Your friends |  | | |  | | | |  | | | |  | | | |  | | |
|  | Your coworkers/colleagues |  | | |  | | | |  | | | |  | | | |  | | |
|  | Your friends in the LGBT community |  | | |  | | | |  | | | |  | | | |  | | |
|  | **When people in all the six categories are considered, how many possess the following assets / resources?** | **All** | | | **Most** | | | | **Some** | | | | **Few** | | | | **None** | | |
|  | Certain political power |  | | |  | | | |  | | | |  | | | |  | | |
|  | Wealth or owners of an enterprise or a company |  | | |  | | | |  | | | |  | | | |  | | |
|  | Broad connections with others |  | | |  | | | |  | | | |  | | | |  | | |
|  | High reputation or are influential |  | | |  | | | |  | | | |  | | | |  | | |
|  | With high school or more education |  | | |  | | | |  | | | |  | | | |  | | |
|  | With a professional job (e.g. lawyers, engineers, doctors, architects etc.) |  | | |  | | | |  | | | |  | | | |  | | |
|  | **How would you rate the number of the following two types of groups/organizations existing in your own community? This may include, but is not limited to the LGBT community.** | **A lot** | | | **More than average** | | | | **Average** | | | | **Less than average** | | | | **A few** | | |
|  | Governmental, political, economic and social groups/organizations (volunteer groups, political interest groups or political parties, social service groups etc) |  | | |  | | | |  | | | |  | | | |  | | |
|  | Cultural, recreational and leisure groups/organizations (religious, alumni, sport, music, dances, crafts, games, etc) |  | | |  | | | |  | | | |  | | | |  | | |
|  | **For how many of these organizations do you participate in activities?** | **All** | | | **Most** | | | | **Some** | | | | **Few** | | | | **None** | | |
|  | Governmental, political, economic and social groups/organizations (political parties, women's groups, village committees, trade union, cooperate associations, volunteer groups, etc) |  | | |  | | | |  | | | |  | | | |  | | |
|  | Cultural, recreational and leisure groups/organizations (religious, country fellows, alumni, sport, music, dances, crafts, games, etc) |  | | |  | | | |  | | | |  | | | |  | | |
|  | **Among each of the two types of groups and organizations in your community, how many represent your rights and interests? This may include, but is not limited to the LGBT community.** | **All** | | | **Most** | | | | **Some** | | | | **Few** | | | | **None** | | |
|  | Governmental, political, economic and social groups/organizations (political parties, women's groups, village committees, trade union, cooperate associations, volunteer groups, etc) |  | | |  | | | |  | | | |  | | | |  | | |
|  | Cultural, recreational and leisure groups/organizations (religious, country fellows, alumni, sport, music, dances, crafts, games, etc) |  | | |  | | | |  | | | |  | | | |  | | |
|  | **Among each of the two types of groups and organizations, how many will help you upon your request?** | **All** | | | **Most** | | | | **Some** | | | | **Few** | | | | **None** | | |
|  | Governmental, political, economic and social groups/organizations (political parties, women's groups, village committees, trade union, cooperate associations, volunteer groups, etc) |  | | |  | | | |  | | | |  | | | |  | | |
|  | Cultural, recreational and leisure groups/organizations (religious, country fellows, alumni, sport, music, dances, crafts, games, etc) |  | | |  | | | |  | | | |  | | | |  | | |
|  | **When all groups and organizations in the two categories above are considered, how many possess the following assets/resources?** | **All** | | | **Most** | | | | **Some** | | | | **Few** | | | | **None** | | |
|  | Significant power for decision making |  | | |  | | | |  | | | |  | | | |  | | |
|  | Financial power |  | | |  | | | |  | | | |  | | | |  | | |
|  | Broad social connections |  | | |  | | | |  | | | |  | | | |  | | |
|  | Great social influence |  | | |  | | | |  | | | |  | | | |  | | |
| **Outness Inventory (Mohr & Fassinger, 2000)** | | | | | | | | | | | | | | | | | | | |
| **Use the following rating scale to indicate how open you are about your sexual orientation to the people listed below. Try to respond to all of the items, but leave items blank if they do not apply to you.**  1 = person definitely does NOT know about your sexual orientation status  2 = person might know about your sexual orientation status, but it is NEVER talked about  3 = person probably knows about your sexual orientation status, but it is NEVER talked about  4 = person probably knows about your sexual orientation status, but it is RARELY talked about  5 = person definitely knows about your sexual orientation status, but it is RARELY talked about  6 = person definitely knows about your sexual orientation status, and it is SOMETIMES talked about  7 = person definitely knows about your sexual orientation status, and it is OPENLY talked about  0 = not applicable to your situation; there is no such person or group of people in your life | | | | | | | | | | | | | | | | | | | |
|  | Mother | 1 | 2 | | | 3 | 4 | | | | 5 | | | 6 | | 7 | | 0 | |
|  | Father | 1 | 2 | | | 3 | 4 | | | | 5 | | | 6 | | 7 | | 0 | |
|  | Siblings (Sisters, Brothers) | 1 | 2 | | | 3 | 4 | | | | 5 | | | 6 | | 7 | | 0 | |
|  | Extended family / relatives | 1 | 2 | | | 3 | 4 | | | | 5 | | | 6 | | 7 | | 0 | |
|  | My new straight friends | 1 | 2 | | | 3 | 4 | | | | 5 | | | 6 | | 7 | | 0 | |
|  | My work peers | 1 | 2 | | | 3 | 4 | | | | 5 | | | 6 | | 7 | | 0 | |
|  | My work supervisor(s) | 1 | 2 | | | 3 | 4 | | | | 5 | | | 6 | | 7 | | 0 | |
|  | Members of my religious community (e.g. church, temple) | 1 | 2 | | | 3 | 4 | | | | 5 | | | 6 | | 7 | | 0 | |
|  | Leaders of my religious community (e.g. church, temple) | 1 | 2 | | | 3 | 4 | | | | 5 | | | 6 | | 7 | | 0 | |
|  | Strangers, new acquaintances | 1 | 2 | | | 3 | 4 | | | | 5 | | | 6 | | 7 | | 0 | |
|  | My old (long-time) heterosexual friends | 1 | 2 | | | 3 | 4 | | | | 5 | | | 6 | | 7 | | 0 | |
|  | General healthcare professionals (e.g. general practitioners, family doctors) | 1 | 2 | | | 3 | 4 | | | | 5 | | | 6 | | 7 | | 0 | |
|  | Sexual healthcare professionals (e.g. specialist sexual health doctors and nurses) | 1 | 2 | | | 3 | 4 | | | | 5 | | | 6 | | 7 | | 0 | |
| **Sexual Risk Behaviors** | | | | | | | | | | | | | | | | | | | |
|  | At what age did you first have a sexual experience involving oral sex? | [Open-ended response] | | | | | | | | | | | | | | | | | |
|  | At what age did you first have a sexual experience involving anal sex? | [Open-ended response] | | | | | | | | | | | | | | | | | |
| **For the remainder of the questionnaire, please refer to the following definitions:**  **•** Regular partner refers to your boyfriend, partner, spouse, or any person whom you are in a long-term sexual relationship with.  • Casual partner refers to a person who is not paid for sex and whom you are not in a long-term sexual relationship with.  • Sex worker / Money Boy refers to a person whom you pay in exchange for sex. | | | | | | | | | | | | | | | | | | | |
| **In the last 6 months, how often did you use a condom when having:** | | **Always** | | **> half the time** | | | | **Half the time** | | | | **< half the time** | | | **Never used a condom** | | | | **N/A** |
|  | Oral sex with a regular partner? |  | |  | | | |  | | | |  | | |  | | | |  |
|  | Anal sex with a regular partner? |  | |  | | | |  | | | |  | | |  | | | |  |
|  | Oral sex with a casual partner? |  | |  | | | |  | | | |  | | |  | | | |  |
|  | Anal sex with a casual partner? |  | |  | | | |  | | | |  | | |  | | | |  |
|  | Oral sex with a sex worker/money boy? |  | |  | | | |  | | | |  | | |  | | | |  |
|  | Anal sex with a sex worker/money boy? |  | |  | | | |  | | | |  | | |  | | | |  |
| **HIV/STI Testing Behaviors** | | | | | | | | | | | | | | | | | | | |
|  | When did you go for you last (most recent) voluntary HIV test? | Never  In the last 6 months  6 to 12 months ago  More than 1 year ago | | | | | | | | | | | | | | | | | |
|  | When did you go for you last (most recent) voluntary test for other STDs or STIs (e.g. Syphilis, Chlamydia, Gonorrhea etc.)? | Never  In the last 6 months  6 to 12 months ago  More than 1 year ago | | | | | | | | | | | | | | | | | |
|  | On average, how regularly do you test for HIV? | I do not test regularly  Once every few years  Once a year  Once every 6 months  Once every 3 months  Once a month  Others (please specify): ______________________ | | | | | | | | | | | | | | | | | |
|  | On average, how regularly did you test for other STDs or STIs? | I do not test regularly  Once every few years  Once a year  Once every 6 months  Once every 3 months  Once a month  Others (please specify): ______________________ | | | | | | | | | | | | | | | | | |
|  | Where do you typically get your voluntary HIV test done? You may choose more than one answer. | Government-Run Clinic (e.g. DSC Clinic)  Government-Run Hospital (e.g. General hospital)  GP Clinic (Regular testing hours)  GP Clinic (Anonymous testing hours)  Anonymous Test Site (NGO: Action for AIDS)  Overseas (please specify where): ______________  Others (please specify): ______________________ | | | | | | | | | | | | | | | | | |
|  | Where do you typically get your voluntary STI test done? You may choose more than one answer. | Government-Run Clinic (e.g. DSC Clinic)  Government-Run Hospital (e.g. General hospital)  GP Clinic (Regular testing hours)  GP Clinic (Anonymous testing hours)  Anonymous Test Site (NGO: Action for AIDS)  Overseas (please specify where): ______________  Others (please specify): ______________________ | | | | | | | | | | | | | | | | | |
|  | In the last 6 months, were you diagnosed with any of the following sexually transmitted infections? | **Please select all that apply:**  HIV  Gonorrhea  Syphilis  Chlamydia  Genital Herpes  Genital Warts  Hepatitis C  Others (please specify): ______________________ | | | | | | | | | | | | | | | | | |
| **HIV Pre-Exposure Prophylaxis and Post-Exposure Prophylaxis** | | | | | | | | | | | | | | | | | | | |
|  | Have you heard of HIV Pre-Exposure Prophylaxis (PrEP)? | Yes, I am currently on PrEP 🡪 go to Q49  Yes, I have taken it but no longer do so 🡪 go to Q49  Yes, I have heard of it but not taken it 🡪 go to Q53  No, I have never heard of it 🡪 skip to Q54 | | | | | | | | | | | | | | | | | |
|  | **If you have taken PrEP**, how long have you been/were you on PrEP? | Less than 3 months  Between 3 to 6 months  Between 6 to 12 months  For more than a year | | | | | | | | | | | | | | | | | |
|  | **If you have taken PrEP**, did you take it daily or on-demand? | **Please select all that apply:**  Daily PrEP  On-Demand PrEP | | | | | | | | | | | | | | | | | |
|  | **If you have taken PrEP**, where did you purchase the medication for PrEP? | **Please select all that apply:**  Government-Run Clinic (e.g. DSC Clinic)  Government-Run Hospital (e.g. General hospital)  GP Clinic  Online supplier  From friends who are currently on PrEP  From friends who are HIV-positive  From local importers of generic drugs  Overseas (please specify where): ______________  Others (please specify): ______________________ | | | | | | | | | | | | | | | | | |
|  | **If you have taken PrEP**, where did you receive your follow-up care for PrEP? | **Please select all that apply:**  Government-Run Clinic (e.g. DSC Clinic)  Government-Run Hospital (e.g. General hospital)  GP Clinic  Overseas (please specify where): ______________  Others (please specify): ______________________  I was able to take PrEP without the guidance of a doctor | | | | | | | | | | | | | | | | | |
|  | **If you have not taken PrEP,** would you consider taking PrEP? | Yes  No | | | | | | | | | | | | | | | | | |
|  | Have you heard of HIV Post-Exposure Prophylaxis (PEP)? | Yes, I have taken it before 🡪 go to Q55  Yes, I have heard of it but not taken it 🡪 go to Q56  No, I have never heard of it 🡪 skip to Q56 | | | | | | | | | | | | | | | | | |
|  | **If you have taken PEP**, where did you receive the treatment for PEP? | **Please select all that apply:**  Government-Run Clinic (e.g. DSC Clinic)  Government-Run Hospital (e.g. General hospital)  GP Clinic  Online supplier  From friends who have been prescribed PrEP/PEP  From local importers of generic drugs  Overseas (please specify where): ______________  Others (please specify): ______________________ | | | | | | | | | | | | | | | | | |
| **Substance Use** | | | | | | | | | | | | | | | | | | | |
|  | Have you ever used any of the following drugs or substances ***during*** sex (i.e. chemsex, chill fun, cf etc.)?? | **Please select all that apply:**  Alcohol  Poppers  Heroin  Crystal Meth (‘Ice’/’Cream’)  Marijuana  Ketamine  Ecstasy  GHB / GBL  Erectile dysfunction medication – e.g. Viagra | | | | | | | | | | | | | | | | | |
|  | Where did you mainly get your drugs (excluding alcohol) from? | **Please select all that apply:**  I have contacts for drug suppliers or pushers  I buy them off my other drug-using friends when I need it  I only use drugs that I get from my sexual partners  I get them from money boys or male sex workers  I go to bars or clubs to look for suppliers  I go to saunas or other sex-on-premises venues to look for suppliers  I bring the drugs back from overseas  Others (Please specify): _________________________ | | | | | | | | | | | | | | | | | |
|  | **In the last 6 months, how often did you use the following substances during sex, or for the purpose of sex?** | **Always** | | | **More than half the time** | | | | **Half the time** | | | | **Less than half the time** | | | | **Did not use it for sex** | | |
|  | Alcohol |  | | |  | | | |  | | | |  | | | |  | | |
|  | Popper |  | | |  | | | |  | | | |  | | | |  | | |
|  | Heroin |  | | |  | | | |  | | | |  | | | |  | | |
|  | Crystal Meth (‘Ice’ / ‘Cream’) |  | | |  | | | |  | | | |  | | | |  | | |
|  | Marijuana |  | | |  | | | |  | | | |  | | | |  | | |
|  | Ketamine |  | | |  | | | |  | | | |  | | | |  | | |
|  | Ecstasy |  | | |  | | | |  | | | |  | | | |  | | |
|  | GHB / GBL |  | | |  | | | |  | | | |  | | | |  | | |
|  | Erectile dysfunction medication – e.g. Viagra, Cialis, Black Ants |  | | |  | | | |  | | | |  | | | |  | | |
|  | How often did you inject any of the above substances in the last 6 months? | Always  More than half the time  Half the time  Less than half the time  Never | | | | | | | | | | | | | | | | | |
|  | How often did you use more than one type of substance at the same time or simultaneously (i.e. mixing drugs or a ‘cocktail’ of drugs) during sex in the last 6 months? | Always  More than half the time  Half the time  Less than half the time  Never | | | | | | | | | | | | | | | | | |
|  | How often did these chemsex sessions include 3 persons or more (i.e. orgies or group sex) in the last 6 months? | Always  More than half the time  Half the time  Less than half the time  Never | | | | | | | | | | | | | | | | | |
| **Single-Item Self-Esteem Scale** | | | | | | | | | | | | | | | | | | | |
|  | I have high self-esteem | 1 = Very Untrue of Me --- 7 = Very True of Me | | | | | | | | | | | | | | | | | |
| **Stigma and Discrimination** | | | | | | | | | | | | | | | | | | | |
| **Perceived Homosexual Stigma (Smolenski, Ross, Risser, & Rosser, 2009)** | | **Strongly Agree** | | **Agree** | | | | | | **Disagree** | | | | | **Strongly Disagree** | | | | |
|  | Many people believe that gay men have psychological problems. |  | |  | | | | | |  | | | | |  | | | | |
|  | Many people do not see gay men as real men. |  | |  | | | | | |  | | | | |  | | | | |
|  | Most families would be disappointed to have a gay son. |  | |  | | | | | |  | | | | |  | | | | |
|  | Many people think that gay men have HIV and will die of AIDS. |  | |  | | | | | |  | | | | |  | | | | |
|  | Many people do not accept same-sex male couples. |  | |  | | | | | |  | | | | |  | | | | |
|  | Many people believe that gay men should not hug, hold hands, or kiss in public. |  | |  | | | | | |  | | | | |  | | | | |
| **Internalized Homosexual Stigma (Amola & Grimmett, 2015)** | | **Strongly Agree** | | **Agree** | | | | | | **Disagree** | | | | | **Strongly Disagree** | | | | |
|  | I have tried to stop being attracted to men. |  | |  | | | | | |  | | | | |  | | | | |
|  | If someone offered me the chance to be completely heterosexual, I would accept the chance. |  | |  | | | | | |  | | | | |  | | | | |
|  | I wish I wasn’t attracted to men. |  | |  | | | | | |  | | | | |  | | | | |
|  | I would like to get professional help in order to be less sexually attracted to men. |  | |  | | | | | |  | | | | |  | | | | |
|  | I feel that being attracted to men is a shortcoming for me. |  | |  | | | | | |  | | | | |  | | | | |
| **Experienced Homosexual Stigma (Bruce, Ramirez-Valles, & Campbell, 2008)** | | **Never** | | **Rarely** | | | | | | **Sometimes** | | | | | **Many times** | | | | |
|  | Growing up, were you made fun of or called names by your own family, because of the way you behaved? |  | |  | | | | | |  | | | | |  | | | | |
|  | Growing up, did other kids make fun of you or call you names because of the way you behaved? |  | |  | | | | | |  | | | | |  | | | | |
|  | Growing up, were you pushed around or beaten up because of the way you behaved? |  | |  | | | | | |  | | | | |  | | | | |
|  | Growing up, did members of your family tell you to change your behavior because you looked too effeminate? |  | |  | | | | | |  | | | | |  | | | | |
|  | As an adult, has your family made fun of you or called you names because of your sexual orientation? |  | |  | | | | | |  | | | | |  | | | | |
|  | Were you rejected by your family because of your sexual orientation? |  | |  | | | | | |  | | | | |  | | | | |
|  | Have you been told to consult a mental health professional because of your sexual orientation? |  | |  | | | | | |  | | | | |  | | | | |
|  | Have you moved away from friends and family because of your sexual orientation? |  | |  | | | | | |  | | | | |  | | | | |
|  | Has a friend rejected you because of your sexual orientation? |  | |  | | | | | |  | | | | |  | | | | |
|  | Have you been treated differently in social situations because of your sexual orientation? |  | |  | | | | | |  | | | | |  | | | | |
|  | Have you been treated unfairly at work because of your sexual orientation? |  | |  | | | | | |  | | | | |  | | | | |
|  | Have you received poor service because of your sexual orientation? |  | |  | | | | | |  | | | | |  | | | | |
|  | Have you been made fun of or called names by other people because of your sexual orientation? |  | |  | | | | | |  | | | | |  | | | | |
|  | Have you been pushed around or beaten up because of your sexual orientation? |  | |  | | | | | |  | | | | |  | | | | |
| **Patient Health Questionnaire-9 (PHQ-9) (Kroenke, Spitzer, & Williams, 2001)** | | | | | | | | | | | | | | | | | | | |
| **Over the last 2 weeks, how often have you been bothered by any of the following problems?** | | **Not at all** | | **Several days** | | | | | | **More than half the days** | | | | | **Nearly every day** | | | | |
|  | Little interest or pleasure in doing things? |  | |  | | | | | |  | | | | |  | | | | |
|  | Feeling down, depressed, or hopeless? |  | |  | | | | | |  | | | | |  | | | | |
|  | Trouble falling or staying asleep, or sleeping too much? |  | |  | | | | | |  | | | | |  | | | | |
|  | Feeling tired or having little energy? |  | |  | | | | | |  | | | | |  | | | | |
|  | Poor appetite or overeating? |  | |  | | | | | |  | | | | |  | | | | |
|  | Feeling bad about yourself – or that you are a failure or have let yourself or your family down? |  | |  | | | | | |  | | | | |  | | | | |
|  | Trouble concentrating on things, such as reading the newspaper or watching television? |  | |  | | | | | |  | | | | |  | | | | |
|  | Moving or speaking slowly that other people could have noticed? Or so fidgety or restless that you have been moving a lot more than usual? |  | |  | | | | | |  | | | | |  | | | | |
|  | Thoughts that you would be better off dead, or thoughts of hurting yourself in some way? |  | |  | | | | | |  | | | | |  | | | | |
| **Suicide Ideation** | | | | | | | | | | | | | | | | | | | |
| Below are some questions on suicide ideation and attempting suicide. We would like to find out more about the prevalence of suicide ideation and attempts in young gay, bisexual, and other men who have sex with men in Singapore and your responses will be kept confidential. However, you may wish to skip this section if it poses any discomfort to you by selecting the option: “Prefer not to say” as a response to any of the questions asked. | | | | | | | | | | | | | | | | | | | |
|  | Have you ever thoughts of attempting suicide (i.e. suicide ideation)? | Yes  No  Prefer not to say (Skip to Q83) | | | | | | | | | | | | | | | | | |
|  | If yes, at what age were you when you had thoughts of attempting suicide? | [Open-ended number]  Prefer not to say (Skip to Q83) | | | | | | | | | | | | | | | | | |
|  | Have you ever attempted suicide? | Yes  No  Prefer not to say (Skip to end) | | | | | | | | | | | | | | | | | |
|  | If yes, did you attempt suicide more than once? | Yes  No  Prefer not to say (Skip to end) | | | | | | | | | | | | | | | | | |
|  | If yes, what was your age during your first suicide attempt? | [Open-ended number]  Prefer not to say (Skip to end) | | | | | | | | | | | | | | | | | |
| We have now come to the end of the survey. Thank you for your participation.  We understand that some questions that were asked that may have prompted further questions on some of the terms used, or caused you discomfort. We believe some of the resources below may be useful for some questions or discomfort that your participation the survey may have raised:  **For questions on HIV prevention and sexual health, you may approach:**  Action for AIDS Singapore ([info@afa.org.sg](mailto:info@afa.org.sg))  Department of Sexually Transmitted Infections Control (DSC) Clinic ([apptdsc@nsc.com.sg](mailto:apptdsc@nsc.com.sg))  **For support or answers to your questions on suicide, you may approach:**  Samaritans of Singapore ([pat@sos.org.sg](mailto:pat@sos.org.sg) or at their 24-hour hotline 1800-221-4444)  **For support or answers to your questions on sexual identity, stigma, or homophobia, you may approach:**  Oogachaga Singapore ([care@oogachaga.com](mailto:care@oogachaga.com) or at their counselling hotline 62262002)  **For support or answers to your questions on substance use and chemsex, you may approach:**  National Addictions Management Service (All addictions helpline: 67326837)  The Greenhouse Singapore ([info@thegreenhouse.sg](mailto:info@thegreenhouse.sg)) | | | | | | | | | | | | | | | | | | | |
